# Antimicrobial resistance pattern of extended-spectrum β-lactamase-producing *Escherichia coli* isolated from fecal samples of piglets and pig farm workers of selected organized farms of India

**DOI:** 10.14202/vetworld.2020.360-363

**Published:** 2020-02-26

**Authors:** Shikha Tamta, Obli Rajendran Vinodh Kumar, Shiv Varan Singh, Bommenahalli Siddaramiah Pruthvishree, Ravichandran Karthikeyan, Ramkumar Rupner, Dharmendra Kumar Sinha, Bhoj Raj Singh

**Affiliations:** 1 Division of Epidemiology, ICAR-Indian Veterinary Research Institute, Bareilly, Uttar Pradesh, India; 2 Division of Bacteriology and Mycology, ICAR-Indian Veterinary Research Institute, Bareilly, Uttar Pradesh, India

**Keywords:** CTX-M gene, India, multidrug resistance, organized farm, piglets, workers

## Abstract

**Background and Aim:**

Extended-spectrum β-lactamase (ESBL)-producing *Escherichia coli* are gradually increasing worldwide and carry a serious public threat. This study aimed to determine the antimicrobial resistance pattern of ESBL-producing *E. coli* isolated from fecal samples of piglets and pig farm workers.

**Materials and Methods:**

Fecal samples from <3-month-old piglets (n=156) and farm workers (n=21) were processed for the isolation of ESBL-producing *E. coli* in MacConkey agar added with 1 µg/mL of cefotaxime. *E. coli* (piglets=124; farm workers=21) were tested for ESBL production by combined disk method and ESBL E-strip test. Each of the ESBL-positive isolate was subjected to antibiotic susceptibility testing. The ESBL-producing *E. coli* were further processed for genotypic confirmation to CTX-M gene.

**Results:**

A total of 55 (44.4%, 55/124) and nine (42.9%, 9/21) ESBL-producing *E. coli* were isolated from piglets and farm workers, respectively. Antibiotic susceptibility testing of the ESBL-positive *E. coli* isolates from piglets and farm workers showed 100% resistance to ceftazidime, cefotaxime, cefotaxime/clavulanic acid, ceftazidime/clavulanic acid, and cefpodoxime. A proportion of 100% (55/55) and 88.9% (8/9) ESBL-positive *E. coli* were multidrug resistance (MDR) in piglets and farm workers, respectively. On genotypic screening of the ESBL *E. coli* isolated from piglets (n=55), 15 were positive for the *bla*_CTX-M_ gene and of the nine ESBL *E. coli* from farm workers, none were positive for the *bla*_CTX-M_ gene.

**Conclusion:**

Although there was no significant difference in isolation of ESBL-producing *E. coli* between piglets and farm workers, the ESBL-positive *E. coli* from piglets showed relatively higher MDR than farm workers.

## Introduction

Treating infections associated with extended-spectrum β-lactamase (ESBL)-producing *Escherichia coli* are becoming difficult since they are capable of hydrolyzing penicillin, broad-spectrum cephalosporins, and monobactams and are often resistant to other antimicrobial classes such as fluoroquinolones, aminoglycosides, and trimethoprim-sulfamethoxazole [1]. The general trend of antibiotic resistance around the world indicates that an overall decline in the total stock of antibiotic effectiveness [2].

In the livestock sector, antibiotics are not only used for the treatment of infections but also for disease prevention and growth promotion [3]. India is one of the largest consumers of antibiotics in the world with 13 billion standard units in 2010 [2]. In India, human and animals live in close proximity which increases the risk of contamination and resistance between them and 95% of adults in India shows β-lactam antimicrobials resistance in Gram-negative bacteria [4,5]. Reports suggest the possibility of transferring ESBL-producing *E. coli* from farm workers to food animal and *vice versa* [6-10]. Multidrug-resistant *E. coli* are reported in food animals and pet animals of India [11-15]. Besides, food animals may act as a reservoir for ESBL-producing strains and foods as a vehicle for the transfer of β-lactam-producing bacteria [16].

This study was conducted to determine the antimicrobial resistance (AMR) pattern of ESBL-producing *E. coli* isolated from fecal samples of piglets and pig farm workers.

## Materials and Methods

### Ethical approval

Ethical approval was not required for this study.

### Sample collection

A cross-sectional study was conducted between August 2016 and May 2017 to sample five government organized pig farms covering three states, namely, Uttar Pradesh, Karnataka, and Tamil Nadu. The sample was collected from five different organized pig farms, namely, Aligarh (41), Bareilly (30), Chennai farm 1 (28), Chennai farm 2(21), and Hassan (36). The fecal samples were aseptically collected with the help of fecal swab (HiMedia, Mumbai, India) directly from piglet’s rectum and in case of piglet farm workers, swabs were distributed to the farmers which were collected in the next morning (n=21) and transported to the laboratory under cold chain for the isolation of ESBL-producing *E. coli*.

### Isolation and identification of ESBL-producing E. coli

Fecal swabs were pre-enriched in buffered peptone water and incubated at 37°C for 4-6 h. Initial screening was done using MacConkey agar supplemented with cefotaxime at 1 mg/L and plates were then incubated at 37°C for 12 h. The purified cultures of presumptive *E. coli* were stored in nutrient agar for identification by biochemical tests (IMViC pattern). For phenotypic identification of ESBL producers, the combination disk method by cefotaxime and ceftazidime with and without clavulanic acid was used [17] and the isolates were further confirmed by Triple ESBL detection Ezy minimum inhibitory concentration (MIC) Strip (HiMedia, India). The isolates were also tested for antibiotic susceptibility pattern with ceftazidime/clavulanic acid (20/10 μg), chloramphenicol (30 μg), cefpodoxime (10 μg), ceftazidime (30 μg), cefotaxime (30 μg), cefepime (30 μg), cefixime (5 μg), cefoxitin (30 μg), piperacillin-tazobactam (100/10 μg), cefotaxime/clavulanic acid (30/10 μg), tetracycline (30 μg), trimethoprim/sulfamethoxazole (1.25/23.75 μg), amikacin (30 μg), and ciprofloxacin (5 μg) by disk diffusion method. The MIC of cefotaxime, cefepime, and ceftazidime was determined by E strips (HiMedia, India). Antimicrobial susceptibility results were interpreted by the following criteria established by the Clinical and Laboratory Standards Institute [18]. *E. coli* showing resistance to at least three classes of antibiotics were categorized as multidrug-resistant strains.

### Genotypic detection of *bla*_CTX-M_ gene

The genomic DNA was extracted from ESBL-positive isolates using QIAamp DNA Mini Kit (Qiagen, Hilden, Germany) and the polymerase chain reaction (PCR) for *bla*_CTX-M_ (forward: CAATGTGCAGCACCAAGTAA; reverse: CGCGA TATCGTTGGTGGTG) was carried out in a final reaction volume of 25 μL [11]. The amplified PCR product was visualized by a gel documentation system (UVP, UK) after electrophoresis in 1.5% (w/v) agarose gel containing ethidium bromide (0.5 µg/mL, Loba Chemie, India). The positive PCR amplicons were sent to commercial sequencing services (Eurofins Ltd., Bangalore) for further purification and sequencing by Sanger method. The homology searches were made using the BLAST algorithm available at http://blast.ncbi.nlm.ni.gov/Blast.cgi, and the representative sequences were submitted to GenBank for accession number.

### Statistical analysis

The association of the isolation of ESBL-producing *E. coli* between piglets and farm workers was tested by Chi-square test/Fisher’s exact (two-tailed) using SPSS version 20.0 statistical software (IBM Corp., Armonk, NY, USA).

## Results

**Figure-1:**
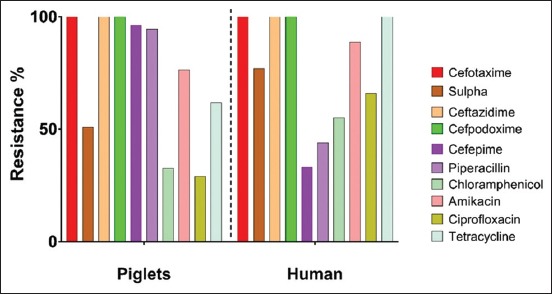
Antimicrobial resistance pattern of extended-spectrum β-lactamase-producing *Escherichia coli* isolates of piglets (n=55) and farm workers (n=9).

A total of 124 and 21 *E*. *coli* were isolated from piglets and human fecal samples, respectively, on MacConkey agar supplemented with cefotaxime. There was no significant difference in isolation of ESBL-producing *E. coli* between piglets and farm workers (p>0.05). Of the 124 isolates from piglets and 21 from farm workers, 55 (44.4%) and 9 (42.9%) isolates were identified as ESBL producers, respectively, by combined disk method. The ESBL-producing isolates from piglets and farm workers had the MIC of cefotaxime >128 µg/mL, cefepime > 32 µg/mL, and ceftazidime> 64 µg/mL. Antibiotic susceptibility testing of the ESBL-positive *E. coli* isolates from piglets (n=55) showed 100% resistance to ceftazidime, cefotaxime, cefotaxime/clavulanic acid, ceftazidime/clavulanic acid, and cefpodoxime. In addition, they were also resistant to cefepime (53, 96.3%), piperacillin (52, 94.5%), amikacin (42, 76.4%) ciprofloxacin (16, 29.1%), chloramphenicol (18, 32.7%), tetracycline (34, 61.8%), and sulfamethoxazole/trimethoprim (28, 50.9%). The ESBL *E. coli* from farm workers showed 100% resistant to tetracycline, ceftazidime, cefotaxime, cefotaxime/clavulanic acid, ceftazidime/clavulanic acid, and cefpodoxime. Further, *E. coli* from farm workers were also resistant to cefepime (3, 33.3%) and piperacillin (4, 44%), amikacin (8, 88.8%), chloramphenicol (5, 55%), sulfamethoxazole/trimethoprim (7, 77%), and ciprofloxacin (6, 66.6%) (Figure-1). A proportion of 100% (55/55) and 88.9% (8/9) ESBL-positive *E. coli* was resistant to more than three classes of antibiotics in piglets and farm workers, respectively, and classified as multidrug resistance (MDR). On genotypic screening of ESBL *E. coli* (n=55), 15 isolates were positive for *bla*_CTX-M_ gene and of the nine ESBL *E. c* oli from farm workers, none were positive for the *bla*_CTX-M_ gene. The representative *bla*_CTX-M_ gene sequences were submitted to GenBank which were assigned with the accession numbers (MF177899, MF 177900).

## Discussion

The emergence and spread of ESBLs producing *E. coli* in food-producing animals is a major public health issue worldwide [11-15]. Piglets and farm workers harbored ESBL *E. coli*, the percentage of cefotaxime and ceftazidime resistance is higher among isolates as compared to ciprofloxacin and chloramphenicol indicates the abundant use of beta-lactam and cephalosporin antimicrobials in piglets and human [5,15]. A recent study documented that the occurrence of ESBL-positive *E. coli* in pigs was not related to total antimicrobial use, but associated with the presence or absence of cephalosporin use at the farm [10]. The current investigations showed that once the bacteria are on the farm or in the farm environment may be widely spread among animals (including insects and rodents) and can reach the environment through manure [19,20]. In this study, the ESBL-producing *E. coli* showed resistance to clinically important antibiotics such as cephalosporins, fluoroquinolones, and aminoglycosides and jeopardize the effective prevention and treatment of various bacterial infections [21]. The present study revealed that all the ESBL-producing E. coli were 100% resistant to cefotaxime and ceftazidime and were concordance with others [11-13]. This study reported that the entire ESBL-positive *E. coli* from piglets as MDR, similarly, in a study from China reported MDR phenotype in total ESBL-producing *E. coli* isolated from pigs [22]. AMR rate depends on the factors around the farm, human behavior, improper sanitation, and hygiene [23]; the improper antibiotic use with route of administration is important for multidrug-resistant bacteria. This study revealed lower resistance for ciprofloxacin, tetracycline, amikacin, and chloramphenicol compared with other antibiotics. The difference might be linked with the overall decline in the use of these antibiotics in India since 2000 [24]. It is also observed that ESBL producers also show resistance to non-β-lactam antimicrobials, such as fluoroquinolones, aminoglycosides, and sulfonamides [22]. The study noticed the CTX-M genotype in ESBL-positive isolates, similar findings were reported in ESBL-producing enterobacterials which might indicate rapid dissemination of *bla*_CTX-M_ genes [25-27]. In ESBL *E. coli*, the most commonly identified enzymes are CTX-M family [28].

## Conclusion

The study highlights the AMR pattern of ESBL-producing *E. coli* isolated from fecal samples of piglets and farm workers. The ESBL-positive *E. coli* from piglets showed relatively higher MDR than farm workers. Hence, necessary steps are needed to reduce the use of antimicrobials in pig farming to decrease the AMR.

## Authors’ Contributions

ORVK, BRS, and DKS conceptualized and designed this research. The research was carried out by ST and BSP. ORVK analyzed the data and result. ORVK, ST, SVS, RK, RR, DKS, and BRS drafted, revised, and finalized the manuscript. All authors read and approved the final manuscript.

## References

[1] Onnberg A, Molling P, Zimmermann J, Soderquist B (2011). Molecular and phenotypic characterization of *Escherichia coli* and *Klebsiella pneumonia* producing extended-spectrum β-lactamases with focus on CTX-M in a low-endemic area in Sweden. APMIS.

[2] Centre for Disease Dynamics, Economics and Policy (2015). Drug Resistance Index and Resistance Map.

[3] Wise R, Hart T, Cars O, Sprenger M.J.W (1998). Antimicrobial resistance. BMJ Clin. Res.

[4] Burch D.G.S (2005). Problems of antibiotics resistance in pigs in UK. Practice.

[5] Walsh T.R, Weeks J, Livermore D.M, Toleman M.A (2011). Dissemination of NDM-1 positive bacteria in the New Delhi environment and its implications for human health:An environmental point prevalence study. Lancet Infect. Dis.

[6] Carattoli A (2001). Importance of integrons in the diffusion of resistance. Vet. Res.

[7] Moodley A, Guardabassi L (2009). Transmission of IncN plasmid carrying bla_CTX-M_-1 between commensal *Escherichia coli* in pigs and farm workers. Antimicrob. Agents Chemother.

[8] Abraham S, Wong H.S, Turnidge J, Johnson J.R, Trott D.J (2014). Carbapenemase-producing bacteriain companion animals:A public health concern on the horizon. J. Antimicrob. Chemother.

[9] Zhang X.L, Wang F, Zhu D.M, Wu S, Wu P.C, Chen Y.D, Wang Y.Q, Zhou L (1998). The carriage of *Escherichia coli* resistant to antibiotics in healthy populations in Shanghai. Biomed. Environ. Sci.

[10] Dohmen W, Bonten M.J.M, Bos M.E.H (2017). Carriage of extended-spectrum β-lactamases in pig farmers is associated with occurrence in pigs. Clin. Microbiol. Infect.

[11] Pruthvishree B.S, Kumar O.R.V, Sinha D.K, Malik Y.P.S, Dubal Z.B, Desingu P.A, Shivakumar M, Krishnaswamy N, Singh B.R (2017). Spatial molecular epidemiology of carbapenem-resistant and New Delhi metallo beta-lactamase (blaNDM) producing *Escherichia coli* in the piglets of organized farms in India. J. Appl. Microbiol.

[12] Pruthvishree B.S, Kumar O.R.V, Sivakumar M, Tamta S, Sunitha R, Sinha D.K, Singh B.R (2018). Molecular characterization of extensively drug-resistant (XDR), extended-spectrum beta-lactamases (ESBL) and New Delhi Metallo beta-lactamase-1 (blaNDM1) producing *Escherichia coli* isolated from a male dog-a case report. Vet. Arh.

[13] Nirupama K.R, Kumar O.R.V, Pruthvishree B.S, Sinha D.K, Murugan M.S, Krishnaswamy N, Singh B.R (2018). Molecular characterization of *bla*_OXA-48_ carbapenemase-, extended-spectrum β-lactamase-and Shiga toxin-producing *Escherichia coli* isolated from farm piglets in India. J. Glob. Antimicrob. Resist.

[14] Murugan S.M, Sinha D.K, Kumar O.R.V, Yadav A.K, Pruthvishree B.S, Vadhana P, Nirupama K.R, Baradwaj M, Singh B.R (2019). Epidemiology of carbapenem-resistant *Escherichia coli* and first report of *bla*_VIM_ carbapenemases gene in calves from India. Epidemiol. Infec.

[15] Kumar O.R.V, Singh B.R, Sinha D.K, Pruthvishree B.S, Tamta S, Dubal Z.B, Karthikeyan R, Rupner R.N, Malik Y.S (2019). Risk factor analysis, antimicrobial resistance and pathotyping of *Escherichia coli* associated with pre-and post-weaning piglet diarrhea in organized farms, India. Epidemiol. Infect.

[16] Overdevest I, Willemsen I, Rijnsburger M, Eustace A, Xu L, Hawkey P (2011). Extended-spectrum β-lactamase genes of Escherichia coli in chicken meat and humans, the Netherlands. Emerg. Infect. Dis.

[17] Andrews J (2012). Detection of extended-spectrum beta-lactamases (ESBLs) in *Escherichia coli* and *Klebsiella* species. J. Antimicrob. Chemother.

[18] Clinical and Laboratory Standard Institute (2014). Performance Standards for Antimicrobial Susceptibility Testing.

[19] Fischer J, Rodriguez I, Schmoger S (2013). *Salmonella enterica* subsp. enterica producing VIM-1 carbapenemase isolated from livestock farms. J. Antimicrob. Chemother.

[20] Guerra B (2013). An Emerging Problem for Public Health:Carbapenemase Producing Microorganisms are also Present in Food Producing Animals, their Environment and Wild Birds. Abstracts of the 5^th^ Symposium on Antimicrobial Resistance in Animals and the Environment, Ghent, Belgium.

[21] World Health Organization (2015). Antimicrobial Resistance, Fact Sheet No 194.

[22] Liu X, Liu H, Wang L, Peng Q, Li Y, Zhou H, Li Q (2018). Molecular characterization of extended-spectrum β-lactamase-producing multidrug-resistant *Escherichia coli* from swine in Northwest China. Front. Microbiol.

[23] Burroughs T, Najafi M, Lemon S.M, Knobler S.L (2003). The Resistance Phenomenon in Microbes and Infectious Disease Vectors:Implications for Human Health and Strategies for Containment:Workshop Summary.

[24] Laxminarayan R, Chaudhury R.R (2016). Antibiotic resistance in India:Drivers and opportunities for action. PLoS Med.

[25] Parajuli N.P, Maharjan P, Joshi G, Khanal P.R (2016). Emerging perils of extended-spectrum β-lactamase producing *Enterobacteriaceae* clinical isolates in a teaching hospital of Nepal. BioMed. Res. Int.

[26] Upadhyay S, Hussain A, Mishra S, Maurya A.P, Bhattacharjee A, Joshi S.R (2015). Genetic environment of plasmid-mediated CTX-M-15 extended-spectrum beta-lactamases from clinical and foodborne bacteria in North-Eastern India. PLoS One.

[27] Ruiz S.J (2011). First characterization of CTX-M-15-producing *Escherichia coli* ST131 and ST405 clones causing community-onset infections in South America. J. Clin. Microbiol.

[28] Adamus W, Baraniak A, Wawszczak M, Głuszek S, Gad B, Wróbel K, Bator P, Majchrzak M, Parniewski P (2018). The genetic background of antibiotic resistance among clinical uropathogenic *Escherichia coli* strains. Mol. Biol. Rep.

